# MicrobiomeStatPlots: Microbiome statistics plotting gallery for meta‐omics and bioinformatics

**DOI:** 10.1002/imt2.70002

**Published:** 2025-02-17

**Authors:** Defeng Bai, Chuang Ma, Jiani Xun, Hao Luo, Haifei Yang, Hujie Lyu, Zhihao Zhu, Anran Gai, Salsabeel Yousuf, Kai Peng, Shanshan Xu, Yunyun Gao, Yao Wang, Yong‐Xin Liu

**Affiliations:** ^1^ Genome Analysis Laboratory of the Ministry of Agriculture and Rural Affairs, Agricultural Genomics Institute at Shenzhen Chinese Academy of Agricultural Sciences Shenzhen China; ^2^ School of Horticulture Anhui Agricultural University Hefei China; ^3^ College of Life Sciences Qingdao Agricultural University Qingdao China; ^4^ Department of Food Science and Nutrition The Hong Kong Polytechnic University Hong Kong SAR China; ^5^ Zhanjiang Key Laboratory of Human Microecology and Clinical Translation Research, the Marine Biomedical Research Institute, College of Basic Medicine Guangdong Medical University Zhanjiang China; ^6^ School of Agricultural Sciences Zhengzhou University Zhengzhou China; ^7^ Jiangsu Co‐Innovation Center for Prevention and Control of Important Animal Infectious Diseases and Zoonoses, College of Veterinary Medicine Yangzhou University Yangzhou China; ^8^ School of Food and Biological Engineering Hefei University of Technology Hefei China; ^9^ School of Ecology and Nature Conservation Beijing Forestry University Beijing China

**Keywords:** bioinformatics, interpretation, microbiome, multi‐omics, visualization

## Abstract

The rapid growth of microbiome research has generated an unprecedented amount of multi‐omics data, presenting challenges in data analysis and visualization. To address these issues, we present MicrobiomeStatPlots, a comprehensive platform offering streamlined, reproducible tools for microbiome data analysis and visualization. This platform integrates essential bioinformatics workflows with multi‐omics pipelines and provides 82 distinct visualization cases for interpreting microbiome datasets. By incorporating basic tutorials and advanced R‐based visualization strategies, MicrobiomeStatPlots enhances accessibility and usability for researchers. Users can customize plots, contribute to the platform's expansion, and access a wealth of bioinformatics knowledge freely on GitHub (https://github.com/YongxinLiu/MicrobiomeStatPlot). Future plans include extending support for metabolomics, viromics, and metatranscriptomics, along with seamless integration of visualization tools into omics workflows. MicrobiomeStatPlots bridges gaps in microbiome data analysis and visualization, paving the way for more efficient, impactful microbiome research.

## Highlights

- Comprehensive visualization resources: MicrobiomeStatPlots provides over 80 reproducible visualization cases and integrates multi‐omics analysis pipelines to facilitate the analysis and interpretation of microbiome data.
- Customizable and user‐friendly: The platform includes detailed tutorials, example datasets, and R‐based plotting codes, enabling researchers to tailor visualizations to their needs.
- Collaborative and open‐source: MicrobiomeStatPlots encourages community contributions to expand its functionality and supports the continuous development of microbiome research. It's accessible for use and contribution at https://github.com/YongxinLiu/MicrobiomeStatPlot.

## Graphical abstract

MicrobiomeStatPlots is a comprehensive platform for microbiome data analysis and visualization, offering bioinformatics tips, integrating multi‐omics pipelines, and featuring 82 visualization and interpretation cases. The platform allows users to customize the code and encourages contributions to expand its content, with plans for future updates. This platform is available at https://github.com/YongxinLiu/MicrobiomeStatPlot.

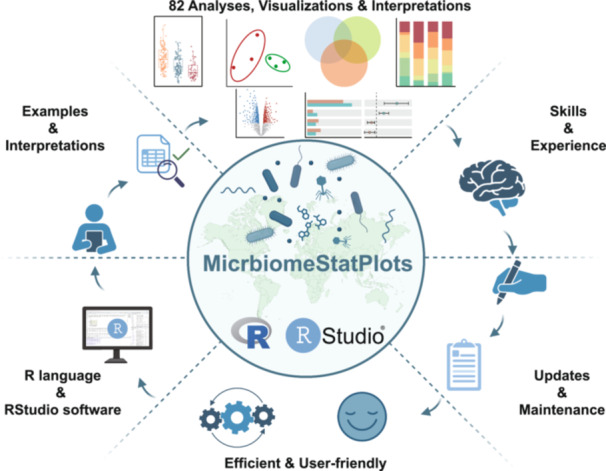

## INTRODUCTION

In recent years, the rapid advancement of omics technologies has revolutionized the characterization of microbial communities, marking the transition to a multi‐omics era for microbiome research [1, 2, 3]. A diverse array of omics approaches, including culturomics, amplicon sequencing, metagenomics, metatranscriptomics, metaproteomics, viromics, and metabolomics, has emerged for studying microbiome [4, 5, 6, 7]. These advancements have driven the development of powerful and user‐friendly bioinformatics tools [8, 9]. The most frequently used tools for amplicon sequencing analysis include QIIME2 [10] and USEARCH [11, 12]. For metagenomic data analysis, commonly used tools include Trimmomatic [13] and fastp [14, 15] for quality control, Kraken2 [16] for accurate taxonomic classification, the HUMAnN3 pipeline [17] for comprehensive functional profiling. Collectively, these innovations have provided vital support for analyzing and exploring various types of omics data.

Omics data analysis has become more accessible over the past few years, with user‐friendly tools and platforms streamlining complex works and enabling users to quickly adapt to data analysis tasks [8, 18, 19, 20, 21, 22]. For example, STAMP [23] is a locally installable software designed for intergroup differential analysis, and provides a variety of visualization options. Pipelines such as Culturome [24], EasyAmplicon [25, 26], and EasyMetagenome (https://github.com/YongxinLiu/EasyMetagenome) utilized for microbial data analysis on local servers, targeting culturome, amplicon, and metagenomic analysis respectively, and providing publication‐ready visualization codes. MetaProteomeAnalyzer [27] is a specialized workflow for analyzing metaproteomics data, and Notame [28] provides a workflow for metabolomics analysis. MicrobiomeAnalyst [8, 18] supports the analysis of amplicons, metagenomics, and metabolomics data, while viromics workflows such as MGV (metagenomic gut virus) [29], ViWrap [30], and Hecatomb [31] are dedicated to virome data analysis. Additionally, comprehensive online platforms like ImageGP [32, 33], OmicsStudio [34], OmicShare tools [35], and Wekemo Bioincloud [4] provide rich and diverse online visualization capabilities. Moreover, some online platforms and databases that target certain aspects (e.g., iNAP [36], GeNets [37], IPGA [38], EVenn [39], and FoodMicroDB [40]) provide users with diversified options for microbiome data analysis and visualization. In addition, some studies have developed R language packages commonly used for omics data analysis [41, 42, 43, 44], while others have reviewed the commonly used R packages and software in omics data analysis and proposed the best analysis practices [45, 46, 47, 48, 49]. Methods for identifying reliable and robust biomarkers from omics data are also rapidly evolving [50, 51, 52, 53]. These advances indicate that multi‐omics analysis has become the cornerstone in addressing scientific questions [5, 6].

Despite these advancements, challenges remain in microbiome data analysis and visualization. Tools like STAMP only focus on a certain type of difference comparison. These tools have limitations in covering a wide range of omics technologies and adapting to rapid iterations in the field. Pipelines or workflows focus on data analysis but lack data visualization in interpretation. Online platforms have a variety of visualization options, but customization is limited. They often lack reproducibility through R code. Some R packages can be used for microbiome data analysis and plotting, but they are relatively scattered and require experience in installing and using these packages. Since R packages are frequently updated, existing code‐based analysis visualization workflows often lack updates, causing some codes fail to run. Comprehensively mastering the basic knowledge and skills of microbiome data analysis is often time‐consuming and laborious. While the primary purpose of visualization is to reduce dimensionality and facilitate result interpretation, existing tools and software often fail to provide adequate interpretation support, leaving users with limited insights. These limitations prevent researchers from efficiently analyzing, visualizing, and interpreting their data.

To address these challenges, we present MicrobiomeStatPlots, a comprehensive resource platform for microbiome data analysis and visualization. MicrobiomeStatPlots provides a collection of 82 R‐based visualization styles designed to meet the diverse needs of multi‐omics researchers. This resource covers culturomics, amplicon sequencing, and metagenomics, while also providing introductory tutorials on bioinformatics essentials, such as Shell and Linux usage, statistical analysis in R, and processing and visualization in Python. Additionally, MicrobiomeStatPlots offers practical guidance on strategies and best practices for microbiome research. MicrobiomeStatPlots stands out by combining basic bioinformatics knowledge with advanced visualization and interpretation tools, making it a valuable platform for both novice and experienced researchers. The project is freely accessible on GitHub (https://github.com/YongxinLiu/MicrobiomeStatPlot), encouraging users to contribute and expand its resources. With ongoing updates and the integration of new visualization tools, MicrobiomeStatPlots aims to support the research community by providing efficient, reproducible, and user‐friendly solutions for microbiome data analysis.

## RESULT

### Overview of MicrobiomeStatPlots

**Figure 1:**
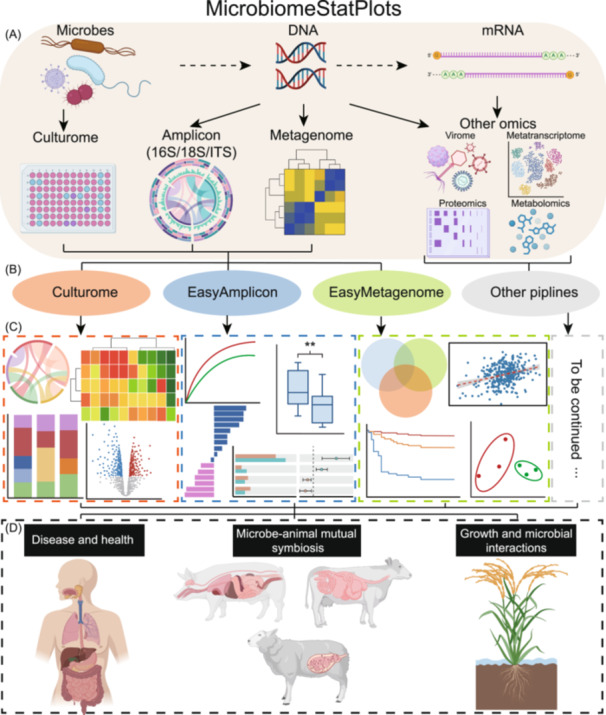
Overview of MicrobiomeStatPlots. (A) Overview of multi‐omics data types analyzed in MicrobiomeStatPlots. The project supports various omics approaches, including culturome, amplicon (16S/18S/ITS), metagenome, and other omics such as virome, metatranscriptome, proteomics, and metabolomics. (B) Core data analysis pipelines included in MicrobiomeStatPlots, such as Culturome, EasyAmplicon, EasyMetagenome, and other pipelines under development. (C) Example visualizations generated by MicrobiomeStatPlots for multi‐omics data analysis. These include heatmaps, box plots, bar plots, scatter plots, and Venn diagrams for different data types, highlighting their applications in understanding microbial communities. (D) Applications of MicrobiomeStatPlots in microbiome research. These include investigations into disease and health, microbe‐animal mutual symbiosis, and plant–microbe interactions, providing insights into the ecological and functional dynamics of microbial communities. Some graphics in this figure are created by BioRender (https://app.biorender.com/).

Microbiome research primarily focuses on microorganisms, DNA, and mRNA. Various omics techniques, such as culturomics, amplicon sequencing, metagenomics, viromics, metabolomics, metatranscriptomics, and proteomics are employed to study microbiome (Figure 1A). MicrobiomeStatPlots extends existing pipelines like Culturome, EasyAmplicon, and EasyMetagenome, by incorporating advanced data statistics, visualization, and result interpretation capabilities. These improvements enable researchers to effectively analyze and understand microbiome data. This platform also supports the development of workflows for additional omics analyses (Figure 1B). MicrobiomeStatPlots offers R code for graphical visualization, a brief introduction to literature sources related to the graphs, and result interpretation, making it easier for researchers to identify and implement appropriate analytical approaches (Figure 1C). MicrobiomeStatPlots supports a wide range of scientific inquiries, including human disease and health, the symbiotic relationship between microbes and animals, and plant‐microbe interactions (Figure 1D). As of 16 January 2025, MicrobiomeStatPlots has gained considerable traction, with 235 forks and 399 stars on its GitHub repository (https://github.com/YongxinLiu/MicrobiomeStatPlot).

### Structure and components of MicrobiomeStatPlots

**Figure 2:**
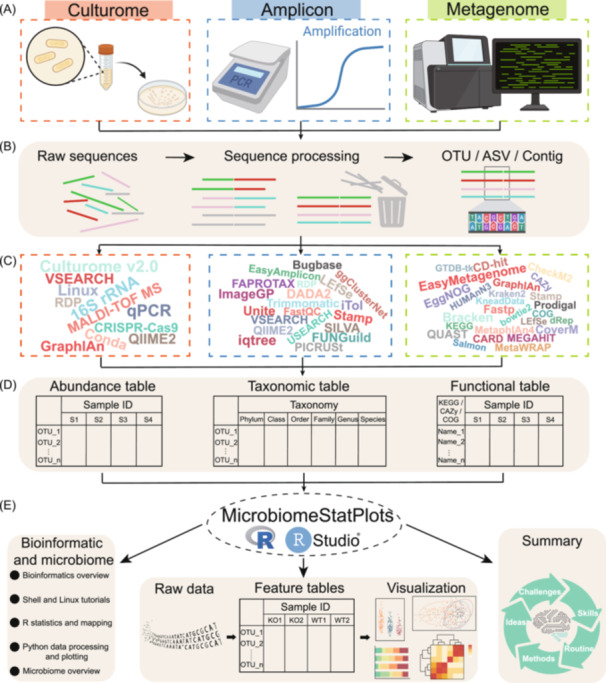
The structure and components of MicrobiomeStatPlots. (A) MicrobiomeStatPlots encompasses the analysis of multi‐omics data, including culturome, amplicon, metagenome, virome, and metatranscriptome, among others. (B) Bioinformatics pipelines for different omics data analysis are included, facilitating the generation of high‐quality raw sequences, sequence processing, and microbiome composition tables. (C) The project is supported by a wide range of constantly updated microbiome‐related software and diverse databases, ensuring robust and reliable data analysis. (D) MicrobiomeStatPlots enables the creation of species composition and functional data tables derived from various omics techniques, streamlining microbiome data processing. (E) The platform integrates foundational bioinformatics knowledge, multi‐omics data analysis pipelines, and methods for interpreting and visualizing omics data. Through continuous updates and iterative improvements, MicrobiomeStatPlots aims to provide comprehensive support for advancing microbiome research. Some graphics in this figure are created by BioRender (https://app.biorender.com/).

In culturomics, amplicon sequencing, or metagenomics, the analysis process starts with raw sequence files, which undergo several sequence processing steps (Figure 2A–B). Using pipelines already developed for culturomics, amplicon sequencing, and metagenomics, in combination with third‐party software analysis (Figure 2C), feature tables are generated to capture microbial abundance, species annotations, and functional characteristics (Figure 2D). Finally, the visualization solutions provided by MicrobiomeStatPlots enable the generation of reproducible plots through R code, and transforming raw data into interpretable figures. This project offers methodological tips for microbiome experimental design, tutorials for using Shell and Linux, statistical analysis in R, and data processing and visualization in Python, creating a one‐stop learning platform for researchers. It aims to support microbiome data analysis, visualization, and interpretation through continuous optimization and sharing (Figure 2E).

MicrobiomeStatPlots systematically organizes knowledge of microbiome data analysis and interpretation in six key areas (Figure 2), including (1) an introductory guide for users, (2) basic instructions for building a microbiome analysis platform on personal computers, and using R software and Linux systems, (3) multi‐omics data analysis pipelines for culturome, amplicon, and metagenome, (4) visualization and interpretation of microbiota feature tables using R and Shell scripts, (5) best practices and innovative approaches for microbiome data analysis, and (6) appendices summarizing other microbiome data analysis techniques. This structured approach ensures that researchers can easily locate and utilize the information they need.

The visualization and interpretation section of MicrobiomeStatPlots is the most frequently updated section and currently includes more than 80 examples of microbiome data analysis, visualization, and interpretation. This section encompasses a range of topics, including the use of base plots (e.g., scatter plots, line plots, and basic bar plots), composite plots (e.g., bubble plots, error bar plots, and Manhattan plots), alpha or beta diversity visualizations (e.g., PCoA, CPCoA, and dbRDA). Additionally, it covers a variety of microbiome data analysis techniques such as difference analysis using MaAsLin2 [54] and ANCOMBC [55], anpan (https://huttenhower.sph.harvard.edu/anpan) for microbial strain assessment [56], and sparse correlations for compositional data (SparCC) co‐abundance network analysis [57]. MicrobiomeStatPlots also includes classification models for identifying microbial biomarkers, such as the random forest model [58], lasso regression using SIAMCAT [59], and the XGBoost model [60]. Detailed explanations and reproducible R codes are provided for each case, enabling researchers to adapt the examples to their specific datasets. To address the problem of frequent updates to R packages or other tools leading to code errors, this project will undergo continuous updates and improvements, like the EasyAmplicon [25, 26]. During this process, R packages and other tools will be regularly updated to maintain compatibility and improve usability, ensuring a more reliable and up‐to‐date platform for users. Additionally, if users encounter code errors, they can report the issue on GitHub (https://github.com/YongxinLiu/MicrobiomeStatPlot/issues), and we will respond promptly to resolve the problem. To further enhance accessibility, these compiled analyses are shared via the “Metagenome” WeChat public account, reaching over 175,000 microbiome researchers. As of January 16, 2025, these shared cases have been viewed more than 40,000 times (Table S1), enabling users to effectively construct and modify combined figures from a variety of omics analyses.

### Case 1: An example for culturome illustration

**Figure 3:**
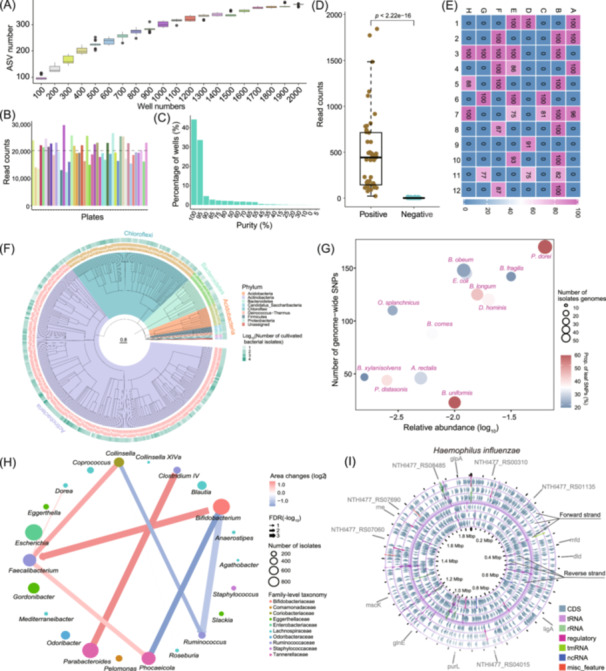
An example of culturome illustration included in MicrobiomeStatPlots. (A) The cumulative curve showed the relationship between the increased well numbers and cultivated amplicon sequence variant (ASV) numbers. (B) The read counts across different plates, with an average of 20,000 reads per plate. (C) The purity distribution of different wells. (D) Comparison for the read counts between negative and positive controls. (E) The purity percentage of cultivated bacteria in a 96‐well plate. (F) Phylogenetic tree of 380 amplicon sequence variants (ASVs). The branch color showed different bacterial phylum. The outer circle shows the relative abundance of each isolated ASVs. (G) An example for the correlation between the relative abundance and number of genome‐wide single‐nucleotide polymorphisms (SNPs) of significantly enriched species. (H) A network visualization of bacterial genera interactions, where red edges represent growth promotion and blue edges indicate inhibition. Edges widths depict interaction significance, while node size corresponds to the number of isolates, and node color reflects taxonomic families. (I) An example of a single bacterial genome of *Haemophilus influenzae*. The reverse and forward strands of the genome of *Haemophilus influenzae* are divided into three reading frames respectively. The three inner circles represent the reverse strand and the three outer circles represent the forward strand. The circle between the reverse strand and the forward strand represents the backbone of the genome.

MicrobiomeStatPlots offers multiple visualization options to display the microbial culture results (Figure 3A–F) update from our previous published Culturome [24]. Box plots illustrate the relationship between the number of culture wells and the number of amplicon sequence variants (ASVs), showing that ASV numbers plateau as the number of wells increases (Figure 3A). Histograms display the microbiota sequence read counts across culture plates, with a dotted line indicating the average read counts approximately 20,000 reads per plate (Figure 3B). Another histogram depicts the distribution of purity percentage across all culture wells, where the horizontal axis represents different purities and the vertical axis indicates the ratio of wells corresponding to different purities; notably over 80% of wells exhibited a purity exceeding 95% (Figure 3C). Box plots compare the read counts of positive and negative controls in cultivation, revealing significantly higher read counts in positive controls (*p *< 0.001) (Figure 3D). A heatmap, plotted using ComplexHeatmap package [61], displays the purity of each well in a 96‐well plate, with darker red and blue colors indicating higher and lower purity levels, respectively (Figure 3E). Furthermore, a phylogenetic tree highlights the composition of cultured microbial species, with species colored by their phylum, and the outer circle indicating the number of cultivate bacterial isolates [62] (Figure 3F). Culturome single‐nucleotide polymorphisms (SNPs) in relation to abundance and the interaction network were also used to further characterize the cultured microorganisms (Figure 3G–H), as referenced in a previous study [63]. The colored bubble plot displays the relationship of the relative abundance of cultivated species and the number of genome‐wide SNPs of each species, with bubble size representing the number of isolate genomes, and different colors indicating the proportion of leaf SNPs (only present in one genotype). This plot reveals a positive correlation between bacterial relative abundance and SNP counts (Figure 3G). An interaction network visualizes the positive and negative interactions between paired‐microbial genera by igraph (https://github.com/igraph/igraph) and ggraph (https://github.com/thomasp85/ggraph). Node sizes represent the number of isolates in each genus, while edges indicate interaction types, with red and blue colors representing positive (growth‐promotion) and negative (growth‐inhibiting) interactions, respectively. The depth of color represents the area (one of morphologic feature of bacterial colony) changes of the bacterial colonies cultured together. The darker the color, the greater the change in area over time. Arrows indicate the direction of interactions. For instance, *Bifidobacterium* promotes the growth of *Faecalibacterium* bacteria but inhibits the growth of *Ruminococcus* (Figure 3H). The effect of genus *Bifidobacterium* on genus *Faecalibacterium* is measured by comparing the colony size (quantified by area) of genus *Faecalibacterium* with and without nearby colonies of genus *Bifidobacterium* [63]. A genome circle map, created using Proksee [64], displays the genome characteristics of *Haemophilus inflenzae*, annotating different types and representative genes (Figure 3I).

### Case 2: An example of amplicon illustration

**Figure 4:**
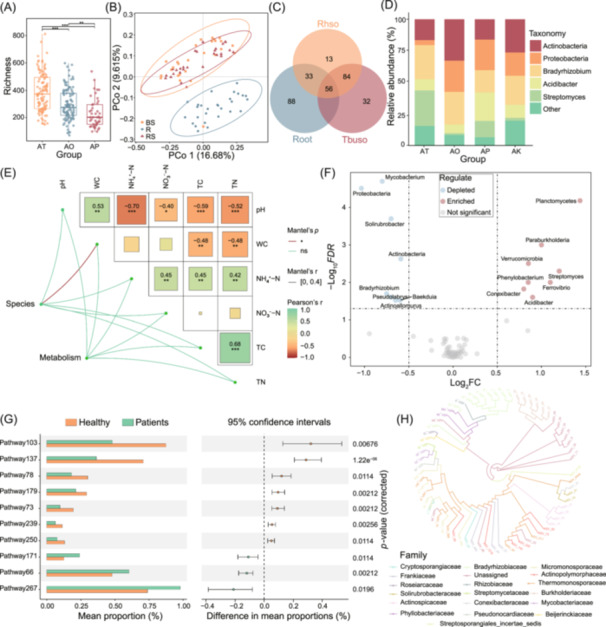
An example of the amplicon illustration included in MicrobiomeStatPlots. (A) Comparison of operational taxonomic units (OTUs) richness among three groups (AT, AO, and AP). (B) Beta diversity analysis showing microbiota community structure differences between groups (BS, R, and RS) using principal coordinate analysis (PCoA). (C) The Venn diagram showing the number of shared and unique OTUs that are the same or different among the three compartments (Root, Rhso, and Tbuso). (D) The stacked bar plot showed the bacteria composition of different groups (AT, AO, AP, and AK) at Phylum level. (E) The Mantel's test heatmap showed the Pearson correlations between soil properties with bacterial species and metabolism. (F) The volcano plot showed the significantly enriched (red) or depleted (blue) genera between different groups. (G) The extended error‐bar plot showed the differences of pathways between patients and healthy groups. The left panel showed the relative abundance in patients (green bar) or healthy (orange bar) groups. The right panel showed the *p‐*value of the differences and 95% confidence intervals. (H) The phylogenetic relationships among different amplicon sequence variants (ASVs), the branches were colored by different families.

In this example, we present eight primary forms of visualization commonly applied in amplicon data analysis, each offering distinct insights into microbial diversity, composition, and relationships. Alpha diversity, which measures the diversity within individual samples (including richness and evenness), is typically illustrated using box plots to compare values between groups. Differences in alpha diversity can be statistically assessed using analysis of variance (ANOVA) (Figure 4A). Beta diversity, which evaluates differences in taxa composition and community structure among samples, is often visualized in scatter plot through principal coordinate analysis (PCoA). This can be plotted using the vegan package (https://github.com/vegandevs/vegan) in R, and differences in beta diversity between groups can be assessed using the permutational multivariate analysis of variance (PERMANOVA) [65, 66] with the “adonis” function in the vegan package (Figure 4B). Venn diagrams are primarily used to assess the quantitative relationships of shared and unique elements among different groups. These can be plotted using the ggVennDiagram package [43] in R software. It is also recommended to use the web version of EVenn (http://www.ehbio.com/test/venn/#/) [49] for plotting (Figure 4C). Stacked bar plots provide an overall visualization of changes in microbial composition between groups and can illustrate species composition at different taxonomic levels. These plots can be generated using the ggplot2 package [67] in R (Figure 4D). Significance correlation heatmap is a statistical tool used to evaluate the strength of relationships between variables. Correlation coefficients are displayed as heatmaps, with color intensity reflecting the strength and proximity of variable associations. The generation of these plots is facilitated by the ggcor or linkET (https://github.com/Hy4m/linkET) packages in R (Figure 4E). Labeled volcano plot is a commonly employed method for displaying significantly upregulated and downregulated genes or species, thereby assisting researchers in the expeditious identification of significant differences in the data (Figure 4F). STAMP plots are frequently employed to illustrate the differential analysis of taxa or functions between groups, exhibiting pairwise comparisons with bar charts, confidence interval distributions, and *p*‐values (Figure 4G). Phylogenetic trees are indispensable for visualizing evolutionary relationships among species. These can be generated using the ggtree package [68, 69, 70] in R (Figure 4H).

### Case 3: An example of metagenome illustration

**Figure 5:**
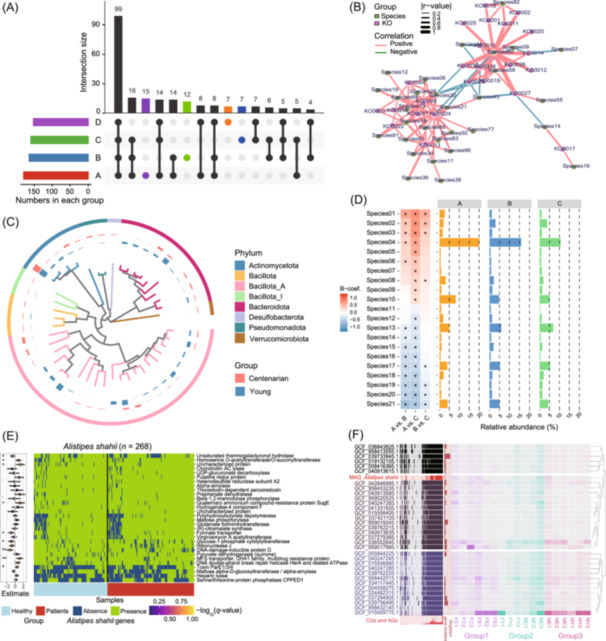
An example of the metagenome illustration included in MicrobiomeStatPlots. (A) An upset plot showed the number of shared and unique species across groups. (B) A co‐abundance network showed the positive and negative correlations between species and KO genes. The correlations and *p* values for each pair were calculated using sparse correlations for compositional data (SparCC) analysis method. (C) A phylogenetic tree showed the phylogenetic relationships of different metagenome‐assembled genomes (MAGs) annotated using human metagenome sequence data. Different phylum levels are marked with different colors. Two circles of histograms outside the branch tree showed the prevalence of each MAG species for young and centenarian group, respectively. (D) Differential analysis of metagenomic species. The left heatmap showed the significance and enriched (red) or depleted (blue) trend between groups. The three combined bar charts on the right showed the relative abundance of each species in each group. (E) The heatmap showed the genes of *Alistipes shahii* associated with the occurrence of disease displaying results using anpan for microbiota strain analysis. The colors showed the absence (blue) or presence (green) of a UniRef90 gene family in metagenomically detected strains for *Alistipes shahii*. The horizontal axis represents samples. The vertical axis shows the functional pathways corresponding to the UniRef90 gene families. The coefficient estimates (dots and error bars on the left) from regression statistics showed the effect of each gene on the outcome (healthy or patient). The different colors of the dots represent different *q* values shown in the legend. Significant effects (*represents *p* < 0.05, **represents *p* < 0.01) are marked with asterisks. (F) The pangenome analysis of *Alistipes shahii* genomes using metagenome sequence data. The heatmap on the left shows the detected (dark areas) or undetected (light areas) of genes in each genome. The lower left bar describes the ratios of core genomes (CGs)/accessory genomes (AGs), the red color represents the ratio of CGs. The horizontal bar chart in the middle shows the number of singleton gene clusters in each genome. Gradient color heatmap on the right depicts genome's median coverage across different samples (Group1 colored in purple, Group2 colored in green, and Group3 colored in red). And all gene clusters are ordered by gene cluster frequency in the genomes of *Alistipes shahii* (cluster tree on the right).

For metagenomic data, MicrobiomeStatPlots offers comprehensive visualization solutions across different aspects (Figure 5). On the one hand, several basic plot types like “Basic bar plot,” “Line plot,” and “Scatter plot” are foundational options. For example, an upset plot can display the intersecting taxa or gene families across groups, highlighting the unique and shared components (Figure 5A). On the other hand, advanced visualization integrates analytical method into the plotting process. For instance, a co‐abundance network illustrates the correlation between species and KO genes, with SparCC method [57] integrated into the result generation process (Figure 5B). A phylogenetic tree circular plot visualizes phylogenetic relationships between different species and compares the relative abundance of each species across groups, and the code for generating the phylogenetic tree circular plot has been integrated into the EasyMetagenome pipeline (https://github.com/YongxinLiu/EasyMetagenome) and the unrooted tree generated using “gtdbtk” method [71] (Figure 5C). A combination of heatmap and histogram displays the differential analysis results between groups and the relative abundance of each species in its corresponding group, and asterisks on the heatmap indicate the significant differences between groups (Figure 5D), and the differential analysis method MaAsLin2 [54] has been integrated into the drawing process. In addition, we also integrated multiple metagenomic data analysis method into MicrobiomeStatPlots. For example, the anpan method (developed by the bioBakery project) (https://github.com/biobakery/anpan) is useful for microbiota strain analysis [17, 56]. MicrobiomeStatPlots provides guidance on using anpan in R, resolving operational errors and enabling gene composition comparisons for strains such as *Alistipes shahii* across groups (Figure 5E). Additionally, pan‐genome analysis using anvi'o [72] enables genome exploration at the species or genus level. Here, we take *Alistipes shahii* as an example to present part of its currently known genomic information (Figure 5F). A step‐by‐step tutorial for pan‐genome analysis is included in MicrobiomeStatPlots, expanding its utility for metagenomic studies.

### Future directions for MicrobiomeStatPlots

**Figure 6:**
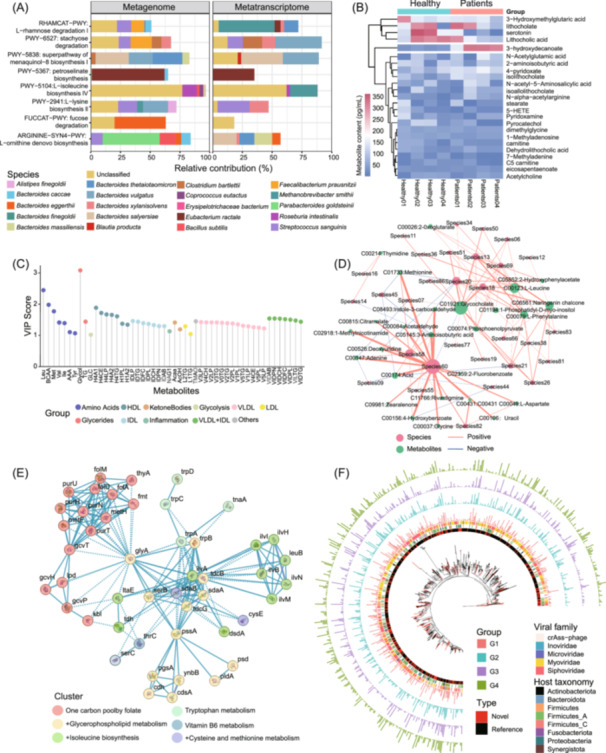
Future outlook of MicrobiomeStatPlots. (A) An example of the comparison of the metabolism results of metagenome and metatranscriptome. The stacked bar plot showed the relative contribution percentage of each species to each pathway. (B) The comparison heatmap of metabolites between healthy and patient groups. (C) The variable influence on projection (VIP) score indicates the importance of each metabolite. Different colors represent different metabolite categories. (D) An example of the spearman correlation network of species and metabolites. The orange and blue colors indicate the positive and negative correlations, separately. The edges width shows the correlation strength. (E) An example of the protein–protein interaction network and grouped in clusters. (F) An example of the phylogenetic tree of different viral operational taxonomic units (vOTUs) and additional features of different groups (G1, G2, G3, and G4). The red leaf on the tree represents the previously undescribed (novel) vOTU. The inner most circle shows the discrimination of novel (red) and reference (previously described) vOTUs. The second circle outward represents different hosts of different vOTUs, and colored according to bacterial phylum level. The third colored circle outward shows the different families of vOTUs. The four outermost circles consisting of bar graphs represent the prevalence of each vOTU in the four groups (G1 colored in red, G2 colored in cyan, G3 colored in purple, and G4 colored in green).

MicrobiomeStatPlots is a comprehensive project for multi‐omics data visualization and interpretation. While the current focus is primarily on culturome, amplicon, and metagenome, there is a growing need to expand the scope to other omics data. The current version of MicrobiomeStatPlots includes limited examples and resources for metabolomics, metaproteomics, viromics, and metatranscriptomics. For example, a horizontal faceted stacked plot is used to compare metabolism results between metagenomes and metatranscriptomes, illustrating compositional percentage differences [73] (Figure 6A). A heatmap highlights metabolite volume differences between healthy and patient groups, clearly showcasing which metabolites undergo drastic changes (Figure 6B). Lollipop plots show the relative importance of each metabolite across groups, offering a visual representation of group‐specific metabolite [74] (Figure 6C). Spearman correlation networks reveal the direction (positive or negative) and strength (indicated by edge width) of correlations between species and metabolites [75] (Figure 6D). A protein–protein interaction network can be generated using the online platforms like STRING (https://string-db.org/) [76, 77], providing insights into protein clusters and interaction types (Figure 6E). A phylogenetic tree displays viral operational taxonomic units (vOTUs) and their respective bacterial hosts across different groups. The outer circles, represented as bar plots, illustrate the prevalence of each vOTU within each group. This figure allows for a clear visualization of the compositional differences of vOTUs among the various groups [78] (Figure 6F). Despite these advances, the project requires significant expansion to fully encompass other omics data. The following future directions are proposed: (1) Develop and incorporate user‐friendly pipelines for metabolomics, metaproteomics, viromics, and metatranscriptomics to broaden the scope of MicrobiomeStatPlots. (2) Compile more examples of data analysis and visualization for various omics types, providing comprehensive resources for interpretation and plotting. (3) Integrate plotting codes directly into omics data analysis pipelines, enabling seamless transitions from feature table generation to visualization and interpretation. For different analysis pipelines, it is essential to first categorize the commonly used data visualization and interpretation cases across various omics. Then, representative analysis methods and visualization styles should be integrated into the appropriate analysis pipelines, with specific modules dedicated to each type of analysis and visualization (e.g., taxa composition overview, differential analysis, and model analysis). (4) Encourage microbiology researchers to contribute to the continuous improvement of data analysis, visualization, and interpretation tools within the project. Increased participations will enhance the project's usability and impact. By addressing these directions, MicrobiomeStatPlots aims to become a more versatile and comprehensive tool for microbiome data analysis, making omics research more accessible and efficient.

## DISCUSSION

MicrobiomeStatPlots provides a reproducible and user‐friendly solution for microbiome data analyzing, plotting, and interpreting, making an important contribution to the visualization and understanding of multi‐omics data. While several analysis platforms have been developed for microbiome data analysis and visualization, such as MicrobiomeAnalyst [18] for metagenomic data analysis, MetaboAnalystR [47] for metabolomic data analysis, and IPGA platform [38] for prokaryotes genomic analysis, these platforms often focus on specific aspect of omics data. Several other data analysis and visualization platforms are widely used in omics research. For example, the Sangerbox platform [79] offers user‐friendly tools for clinical bioinformatics, while the Majorbio Cloud [20] provides multi‐omics data analysis and visualization. ImageGP [32, 33] is popular for microbiome data analysis, and Wekemo Bioincloed [4] offers 22 workflows and 65 visualization tools. However, these platforms still have limitations, such as restricted functionalities for paying to access for some features in some platforms, limited interpretative support, and the lack of customizable, reproducible code. MicrobiomeStatPlots addresses these gaps by providing over 80 multi‐omics data visualization and interpretation cases, covering culturome, amplicon sequencing, metagenome sequencing, and initial examples for metabolomics, metatranscriptomics, metaproteomics, and viromics. This project not only offers visualization but also integrates detailed case interpretations, reproducible codes, and accessible example datasets, allowing users to adapt and expand the analysis.

Moreover, one of the distinguishing features of MicorbiomeStatPlots is its detailed explanations of plotting schemes and examples. This approach complements the shortcomings of existing platforms by providing comprehensive guidance for reproducing figures and interpreting results. Users can access original codes and example data tables, facilitating reproducibility and expansion of the visualizations. These features resolve common pain points, such as the lack of drawing data and data format inconsistencies that hinder rapid figure reproduction. The project also includes basic tutorials on R software and Linux systems, addressing barriers for users unfamiliar with these tools. This knowledge base ensures that the analysis, plotting, and interpretation of microbiome data become more accessible to a broader audience. MicrobiomeStatPlots stands out from other published online platforms in terms of its comprehensive coverage of omics data, the availability of plotting data and code, as well as the presentation and interpretation of visualizations. It offers significant advantages in these areas, making it a valuable complement to existing tools.

Although this study provides numerous examples of microbiome visualization and interpretation, several limitations remain. One limitation is the absence of user‐friendly graphical interfaces or interactive tools for adjusting visualizations. This could present challenges for users who are less familiar with programming or those seeking more intuitive ways to tailor plots to their specific research needs. In the future, as we accumulate more user‐friendly visualization cases, a simpler and more accessible interactive interface might be needed to assist users who are not experienced with programming. Alternatively, these existing visualization cases could be integrated into interactive platforms such as Wekemo Bioincloud [4] and ImageGP [33]. Additionally, while we provide brief introductions and preliminary interpretations for most visualizations and examples in the published paper, the depth of interpretation remains limited. In future updates, we aim to provide a more comprehensive analysis, explaining the roles of various figures in microbiome research and guiding users on how to interpret the elements within these figures. We also plan to provide more detailed explanations of each step in the analysis code. Furthermore, although some integrated visualization and interpretation tools exist in pipelines like EasyAmplicon and EasyMetagenome, a detailed comprehensive framework for seamlessly incorporating these visualization tools into broader research pipelines is still lacking. Moving forward, we plan to organize and classify current microbiome analysis methods and visualization cases, incorporating more accessible tools into established data analysis workflows. To expand the international user base, MicrobiomeStatPlots offers bilingual descriptions in both Chinese and English for each visualization and interpretation case, as well as for the user guide on GitHub (https://github.com/YongxinLiu/MicrobiomeStatPlot). However, much of the content is still written in Chinese. To further broaden the reach of MicrobiomeStatPlots, we will work on translating and sharing these Chinese materials, which will help increase the platform's international visibility and user engagement.

Looking forward, MicrobiomeStatPlots will continue to evolve by incorporating more cases for metabolomics, metatranscriptomics, metaproteomics, and viromics data. The analysis, visualization, and interpretation of culturome, amplicons, and metagenomes will also be further expanded. Moreover, the integration of code cases into existing pipelines such as Culturome [24], EasyAmplicon [25, 26], and EasyMetagenome (https://github.com/YongxinLiu/EasyMetagenome) [80, 81] pipelines will enable users to extract data directly from raw data tables for seamless plotting. Increased community participation and collaboration will further solidify MicrobiomeStatPlots as a comprehensive and adaptable resource for microbiome research.

## CONCLUSION

In summary, MicrobiomeStatPlots offers valuable solutions for the visualization and interpretation of rapidly growing multi‐omics datasets, especially for culturome, amplicon, and metagenome data. Future iterations of the project aim to include more resources for other omics data such as metabolomics, metaproteomics, viromics, and metatranscriptomics. This integration project incorporates three widely used omics data analysis pipelines (culturome, amplicon, and metagenome), more than 80 examples for the analysis and interpreting of multi‐omics data and an updated best practice pipeline for microbiome data analysis. By providing reproducible data analysis and referenceable interpretations of results, the MicrobiomeStatPlots project can provide a personalized solution for microbiome data analysis and interpretation.

## METHODS

MicrobiomeStatPlots is designed as a code‐based framework, employing R software and Shell scripts on a Linux system. For multi‐omics data analysis, various software tools and platforms specifically tailored for microbiome research were used. Since feature tables must be obtained for each omics type, upstream data analysis pipelines are executed before downstream data processing and interpretation. The main steps for culturome, amplicon, and metagenome data analysis can be summarized as follows: For culturome analysis, key steps include preparing mapping files, merging paired‐end sequences, sample splitting, sequencing depth calculation, primer excision, ASV construction, species annotation, and phylogenetic tree generation [24]. Amplicon sequencing analysis involves file preparation, read merging, primer trimming, OTU or ASV clustering, feature table creation, diversity calculation, and species annotation [25, 26]. Metagenome analysis includes data preprocessing, read‐based and assembly‐based analysis, sample binning, and bacterial genome analysis (https://github.com/YongxinLiu/EasyMetagenome) [80, 81].

Numerous R packages were utilized for processing feature tables, plotting and interpreting results. A comprehensive list of the packages used for each analysis or plotting example was provided in Table S2. To date, more than 190 R packages have been used in the visualization and interpretation part of the MicrobiomeStatPlots. The plotting results were mainly interpreted with reference to examples from published papers. Each case contains several parts: (1) A brief introduction to the analysis or plots, including explanations of their composition elements and their roles in representing microbiome data. (2) At least one example from published literatures with detailed explanations of legends and results was provided. (3) Reproducible analysis and plotting codes, along with sample data were provided to ensure seamless operation of the code. The inclusion of original codes and sample datasets enabled users to adapt their data for consistent and reproducible plotting. To assist users with limited experience in R or Linux, the “Bioinformatics_and_microbiome” section included tutorials on the use of R software and Linux system basics, ensuring accessibility and broad usability for microbiome data analysis, plotting, and interpretation.

## AUTHOR CONTRIBUTIONS

**Defeng Bai**: Writing—review and editing; writing—original draft; formal analysis; validation; visualization; methodology; data curation. **Chuang Ma**: Methodology; validation; visualization; writing—original draft; writing—review and editing; formal analysis; data curation. **Jiani Xun**: Methodology; validation; visualization; writing—original draft; formal analysis; data curation; writing—review and editing. **Hao Luo**: Validation; writing—review and editing; software; methodology. **Haifei Yang**: Methodology; software; writing—review and editing; validation. **Hujie Lyu**: Methodology; software; writing—review and editing; validation. **Zhihao Zhu**: Methodology; software; writing—review and editing; validation. **Anran Gai**: Methodology; software; writing—review and editing; validation. **Salsabeel Yousuf**: Methodology; software; writing—review and editing; validation. **Kai Peng**: Methodology; software; writing—review and editing; validation. **Shanshan Xu**: Methodology; validation; writing—review and editing; software. **Yunyun Gao**: Methodology; software; validation; writing—review and editing; funding acquisition. **Yao Wang**: Methodology; validation; writing—review and editing; writing—original draft; software; data curation; project administration. **Yong‐Xin Liu**: Writing—review and editing; funding acquisition; methodology; validation; software; data curation; project administration; conceptualization; supervision; writing—original draft.

## CONFLICT OF INTEREST STATEMENT

The authors declare no conflicts of interest.

## ETHICS STATEMENT

No ethics approval was required for this study because all samples used were publicly available and obtained from open‐access sources.

## Supporting information


**Table S1.** MicrobiomeStatPlots WeChat public account push statistics.

**Table S2.** R software packages used in visualization and interpretation cases in MicrobiomeStatPlots.

## Data Availability

The data that supports the findings of this study are available in the supplementary material of this article. MicrobiomeStatPlots is freely available, implemented in Shell and R, and is easy to download and use. This project is provided on GitHub https://github.com/YongxinLiu/MicrobiomeStatPlot. All figure data can be accessed via GitHub or in the supplementary tables. All the R packages currently in use can be accessed via https://pan.baidu.com/s/1wKSPuOZueJ0H_EsNsnjBYg?pwd=ze7q. Supplementary materials (tables, graphical abstract, slides, videos, Chinese translated version and updated materials) may be found in the online DOI or iMeta Science http://www.imeta.science/.
